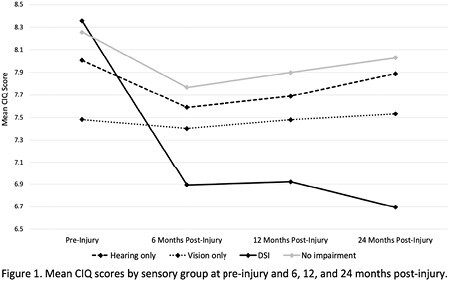# 76 Social Reintegration After Burn Injury Is Negatively Impacted by Sensory Impairments

**DOI:** 10.1093/jbcr/irae036.068

**Published:** 2024-04-17

**Authors:** Maiya I Pacleb, Xinyao deGrauw, Caitlin M Orton, Jeffrey C Schneider, Haig A Yenikomshian, Barclay T Stewart

**Affiliations:** UW Medicine Regional Burn Center, Harborview Medical Center, Seattle, Washington; University of Washington, Seattle, Washington; Spaulding Rehabilitation Hospital/Harvard Medical School, Boston, MA; University of Southern California, Los Angeles, CA; UW Medicine Regional Burn Center, Harborview Medical Center, Seattle, Washington; University of Washington, Seattle, Washington; Spaulding Rehabilitation Hospital/Harvard Medical School, Boston, MA; University of Southern California, Los Angeles, CA; UW Medicine Regional Burn Center, Harborview Medical Center, Seattle, Washington; University of Washington, Seattle, Washington; Spaulding Rehabilitation Hospital/Harvard Medical School, Boston, MA; University of Southern California, Los Angeles, CA; UW Medicine Regional Burn Center, Harborview Medical Center, Seattle, Washington; University of Washington, Seattle, Washington; Spaulding Rehabilitation Hospital/Harvard Medical School, Boston, MA; University of Southern California, Los Angeles, CA; UW Medicine Regional Burn Center, Harborview Medical Center, Seattle, Washington; University of Washington, Seattle, Washington; Spaulding Rehabilitation Hospital/Harvard Medical School, Boston, MA; University of Southern California, Los Angeles, CA; UW Medicine Regional Burn Center, Harborview Medical Center, Seattle, Washington; University of Washington, Seattle, Washington; Spaulding Rehabilitation Hospital/Harvard Medical School, Boston, MA; University of Southern California, Los Angeles, CA

## Abstract

**Introduction:**

Social reintegration is an important goal of recovery for people living with a burn injury. Sensory impairments are common after major burn injury and can compound challenges related to burn recovery more generally. Therefore, we aimed to describe social integration outcomes of people living with burn injury who have hearing, vision, or dual sensory impairment (DSI, hearing and vision impairments) when compared to those with no sensory impairment (NSI). We hypothesized that those with sensory impairments would have lower social integration when compared to those without.

**Methods:**

Adult participants in a multicenter national longitudinal database who provided responses for the abbreviated Community Integration Questionnaire (CIQ) were included. CIQ evaluates social integration on a scale of 0 (no community integration) to 12 (excellent community integration). Participants self-reported (yes/no) sensory impairment at discharge (pre-injury recall) and 6, 12, and 24 months after injury and were then grouped into cohorts with hearing impairments, vision impairments, DSI, and NSI. Mixed effect regression models evaluated the impact of sensory impairments on CIQ compared to pre-injury scores and the differences between cohorts at each timepoint.

**Results:**

852 participants were analyzed, with older participants (≥55 years) more likely to have sensory impairment (p < 0.001). There were 11% (n=95) with hearing impairment, 9% (n=72) with vision impairment, 3% (n=30) with DSI, and 77% (n=655) with NSI. Those with vision impairment reported a significantly lower CIQ score pre-injury compared to those with NSI (p < 0.01). Compared to pre-injury, participants with DSI had significantly lower CIQ at 6, 12, and 24 months (p < 0.01), and participants with NSI had lower CIQ at 6 and 12 months (Figure 1, p< 0.01). Compared to those with NSI, participants with DSI had more CIQ score loss at all follow-up timepoints (0.97 at 6 months, p< 0.05; 1.07 at 12 months, p< 0.05; 1.43 at 24 months, p< 0.01). The CIQ for those with DSI continued to decrease 24 months post-injury, whereas those with hearing impairment, vision impairment, and NSI improved.

**Conclusions:**

While all groups’ social integration decreased after burn injury, significantly lower CIQ scores were reported by those with DSI. Importantly, those with hearing impairment, vision impairment, or NSI had CIQ scores that gradually improved over time. Future studies should evaluate the effect of systematic screening and delivery of key resources for those with sensory impairments after burn injury to aid their community reintegration.

**Applicability of Research to Practice:**

Those who experience sensory impairments after burn injury may need focused rehabilitation efforts and adapted recovery resources that address their unique health and social needs to achieve a successful reintegration.